# Proximal auditory AR rehabilitation: system integration and wellness applications—from hearing support up toward vestibular rehabilitation

**DOI:** 10.3389/fresc.2024.1288945

**Published:** 2024-07-24

**Authors:** Jinyoung Lee, Shigekazu Ishihara, Keiko Ishihara, Ken Ito

**Affiliations:** ^1^ Graduate School of Interdisciplinary Information Studies, The University of Tokyo, Tokyo, Japan; ^2^ Faculty of Health and Wellness Sciences, Hiroshima International University, Hiroshima, Japan

**Keywords:** auditory AR, proximity, extrapyramidal tract, Parkinson’s disease, vestibular rehabilitation

## Abstract

We present a new rehabilitation system based on novel principles, which consists of an auditory augmented reality (AR) headset we originated. The auditory AR headset, which does not cover both ears, allows users to hear both Real and Virtual environmental sounds at the same time. It can also be used in combination with Hearing Aids. We have studied a system to support hearing-impaired people and conducted a test evaluation. The system was able to provide convenience akin to “reading glasses for sound” to those who had mild hearing disabilities. Furthermore, by combining the system with surrounding speakers, a completely novel virtual auditory illusion was created in which the sound image jumps into the ear and runs away. We name this “proximal auditory AR (PAAR)” system. This system directly affects the unconscious level of reflexes for maintaining a standing position and can generate very subtle body motion disturbance. Using this system, we can modulate the standing posture and observe the autonomic nerve system's ability to subliminally compensate for the disturbance, using a stabilometer that measures body sways by center of pressure (COP). We observed a significant difference in the declination of COP only when using the PAAR, which is combined with array speakers and the auditory AR headphone, compared using a conventional closed-type and a bone-conduction headphone. By analyzing such big data of physical movement through machine learning, we expect to realize new systems for diagnosis, rehabilitation, function maintenance, and fall prevention.

## 1 Introduction: genesis of vestibular rehabilitation by auditory AR headset

Augmented reality (AR) was invented from the extension of virtual reality (VR), but its establishment in the 2020s is exclusively in visual applications. Although AR glasses have become popular, especially in industrial settings, AR technology targeting the auditory sense was virtually non-existent, as the auricle could not be covered for safety reasons.

**Figure 1:**
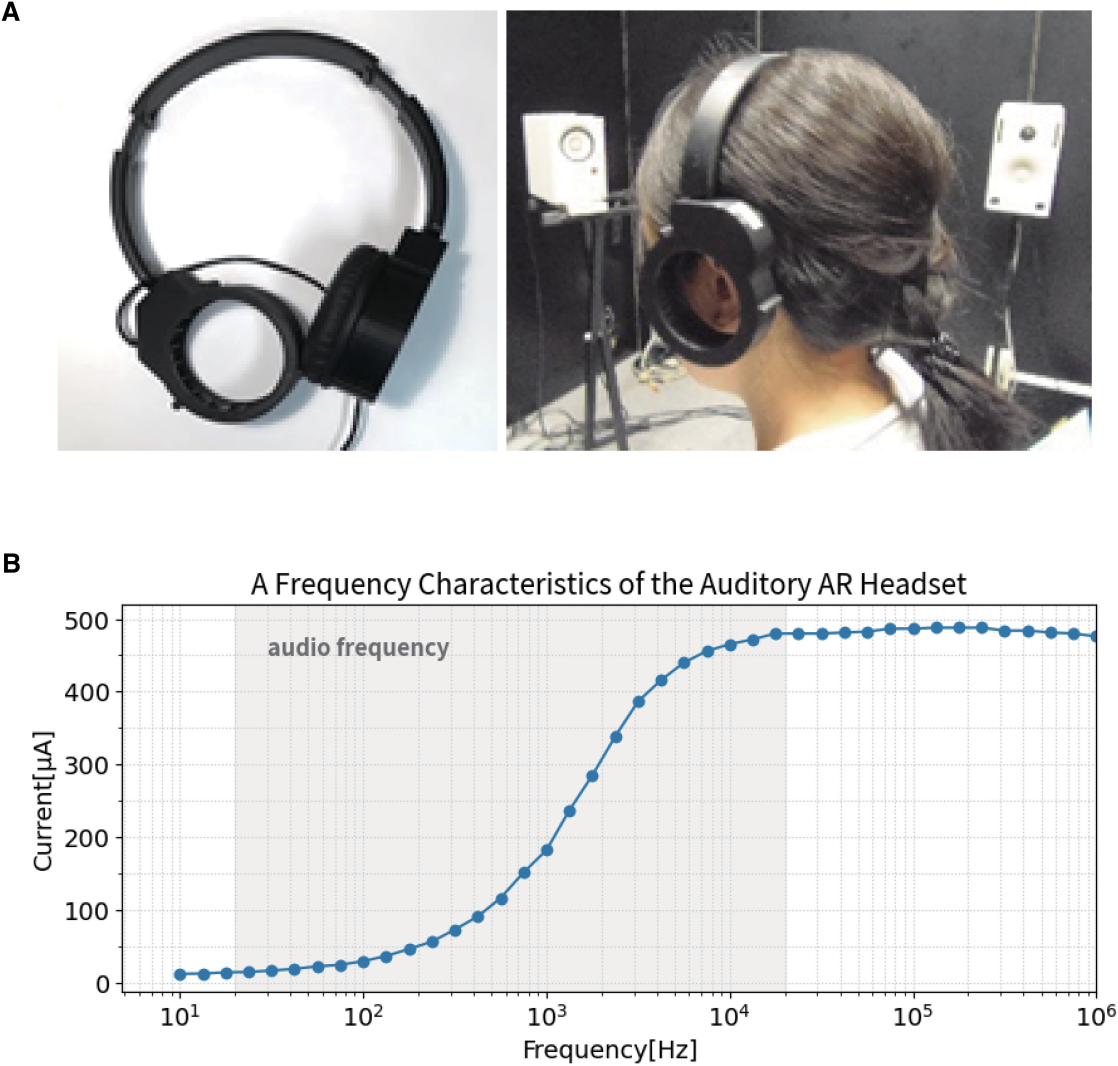
(**A**) Ears-free auditory AR headset. (**B**) A frequency characteristic of the auditory AR headset.

We have solved this problem by developing a full-ears-free headset since 2016 (Figure 1A) (1, 2). Since it does not touch the auricle and does not cover the ear, the wearer can hear sounds in real and virtual space simultaneously. This allows one to receive sound guide information while coping with physical risks in factories.

The auditory AR headset, equipped with a piezoelectric actuator as the sound oscillator, has high-pass filter-like characteristics that enhance the audible band above 1,000 Hz (Figure 1B). Therefore, the headset was expected to assist language listening with minimal energy. Also, because the AR headset does not cover the auricle, it can be used alongside hearing aids worn by the hearing impaired. When the actuator was tested on hearing-impaired people, it was found to provide “reading glasses”-like convenience for those with mild hearing impairment. However, the ears-free auditory AR headphone had limits to improve the severely hearing impaired people, because of its amplitude limit and frequency characteristics. We thus developed another system called proximal auditory AR (PAAR), in which features of the auditory AR headphones would stand out.

Since it does not cover the auricle, it can be used in combination with array speakers. This system reproduces a continuous sound between remote-field and proximal-field virtual direct sound, creating a virtual sound image space in which the sound approaches and jumps into and out of the ear, especially inside and outside the head. This feature was utilized to construct a new vestibular rehabilitation system.

In this article, we propose a wellness application using a “proximal auditory AR” system based on a “full-ears-free headphone.” First, an overview of the system is presented, followed by participant measurements of vestibular function training. The system artificially induces a very slight “body sway,” and uses this artificial sway for functional training of standing posture holding.

A bone-conduction headphone has recently become popular. It vibrates the skull, and because the signal is directly connected to the inner ear, the speed of sound transmission is fast, in the order of milliseconds. Therefore, the virtual impact of the binaural signal is small. This virtual impact is discussed in the following.

Attempts have been made to create a new vestibular rehabilitation system that works on the extrapyramidal system by subliminal stimulation of involuntary movements for standing and body balance.

## 2 Method

### 2.1 System setup

Two speakers are placed 100 cm apart from the midline on either side. This presents the participant with far-distant sounds approaching from a remote place. The arrayed speakers placed to the right and left were adjusted to a height of 150 cm, with the vibrating surfaces facing each other so that the sound would reach the participant's ears perpendicularly. For the near-directed sound, three headphones were used: bone-conduction headphone, conventional closed-type headphone, and ears-free headphone. The combinations are described in Section 2.2. A stabilometer was placed between the left and right array speakers, and the participants were asked to stand on it with wearing each headphone. For the spatial sound operation Spat 5.0 for MAX system by IRCAM (French National Institute for Research and Coordination in Acoustics/Music, Paris, France) is used. We use a Wii Balance Board as a stabilometer to measure body sways by center of pressure (COP), which is produced by Nintendo (Kyoto, Japan). The measurement software for recording body sway data from the stabilometer was WBBSS (Wii Balance Board Stabilometry System), which is published by Assistant Professor Yuki Hyohdoh of Kochi University (3).

#### 2.1.1 Stimuli

For the stimulus sound source, a jingle with an attack was used to induce a sense of spatial localization in the listener. Specifically, a small metallic cymbal sound used in Tibetan Buddhism was processed to 1,000 ms by extracting only the vicinity of the attack.

#### 2.1.2 Process

Participants wore headphones and stood on a stabilometer at the center of the array speakers. The stimulus was the aforementioned jingle (1,000 ms long). This was alternately panned from left to right and right to left five times, for a total of 10 panning sessions per trial, to measure the participant's body sway. Two trials of measurements were taken per participant. There was a 5-s pause between panning to avoid influencing the measurement of body sway that appeared after the first-panning and the second panning.

### 2.2 Measuring conditions

In all measurements, the participant's eyes were opened to prevent falling, and a gazing point was placed to prevent swaying of the body due to eye movement. Under the following five conditions, the body sways were measured.

(1) Silent condition: The body sway measurement was performed for 60 s without any signal.
(2) Array speakers ONLY: The body sway measurement was performed using only far-field acoustic stimuli from speakers array placed to the right and left without wearing headphones that present near-field acoustic stimuli.
(3) Bone-conduction headphone: A combination of bone-conduction headphone and array speakers presents each near-directed and far-directed acoustic stimulus.
(4) Conventional closed-type headphone: A combination of conventional closed-type headphone and array speakers presents each near-directed and far-directed acoustic stimulus.
(5) Ears-free headphone (auditory AR headset): A combination of ears-free headphone and array speakers presents each near-directed and far-directed acoustic stimulus. From the following, the ears-free headphone refers to the auditory AR headphone developed in our laboratory.

### 2.3 Procedure

#### 2.3.1 Participants

A total of 30 students from the Faculty of Rehabilitation at Hiroshima International University cooperated in the study. Of them, 22 were men and 8 were women.

We analyzed the data of 29 participants in Section 3.2, excluding the data for 1 woman, which was a statistical outlier. This participant's body swayed significantly even when no stimulation was applied: In the box plot of the lateral sway component, the maximum value was 7.62 mm, but her value was 9.42 mm.

#### 2.3.2 Sound setting

The presentation of near-directed acoustic stimuli is not always the same in terms of the actual volume reproduced, even if the sound source and sound level on the system side are standardized, owing to the differences in the shape of the headphone and the way the vibration is transmitted. Therefore, in this measurement, we asked six participants to cooperate, adjusted the volume of each headphone by subjective sensory volume, and applied the mean value. The set sound pressure value on the system (PC) side that outputs the signal is described in the following as dB*SSL* (system sound level). The sound pressure level adjusted by the sensory volume was measured with a sound level meter and is denoted as dB*SPL* (sound pressure level).

Since bone-conduction is a different method of transmitting sound than close-type or ears-free headphone, comparisons are difficult. The array speakers were adjusted by subjective sensory volume to provide the most panning. Table 1 provides the output from the system (PC) side as dBSSL (system sound level) and the measured values from the sound level meter as dBSPL (sound pressure level).

**Table 1:** System Sound Level (SSL) adjusted by subjective sensory volume and its physical Sound Pressure Level (SPL).

(a) Array Speakers only			
	Background	Standard stimuli	
System sound level (PC) (dB*SSL*)	—	−20.0	−30.0
Sound pressure level (dB*SPL*)	52.0	86.5	76.7
(b) Standard stimuli with combination of array speakers and headphones			
	Array speakers	Bone-conduction	
System sound level (PC) (dB*SSL*)	−30.0	0.0	
Sound pressure level (dB*SPL*)	—		
	Array speakers	Conventional closed-type	
System sound level (PC) (dB*SSL*)	−40.0	0.0	
Sound pressure level (dB*SPL*)	63.7		
	Array speakers	Ears-free auditory AR	
System sound level (PC) (dB*SSL*)	−45.0	−35.0	
Sound pressure level (dB*SPL*)	63.2		

From these measurements, it can be seen that the sound pressure presented by the conventional closed-type headphone (63.7 dB*SPL*) and the sound pressure presented by our ears-free headset (63.2 dB*SPL*) are almost the same in the physical sound pressure measurements. In the following, we will evaluate and discuss the results of the participant measurements under these conditions.

#### 2.3.3 Measurement

First, all participants were measured for the body sway in the silent condition and with array speakers only. In the case of bone-conduction headphone, conventional closed-type headphone, and ears-free headphone, six (3!) different orders of measurement were prepared to avoid hysteresis bias, and five participants were measured in each order, following which the results were averaged. In the case of bone-conduction headphone, conventional closed-type headphone, and ears-free headphone, the measurements were taken twice each.

## 3 Results

### 3.1 Body sway measurement

#### 3.1.1 Probability elliptic evaluation method

In this measurement, alternating left–right panning sound stimuli were presented with a bone-conduction headphone, a conventional closed-type headphone, and an ears-free headphone to examine their effects on body sway. It is difficult to evaluate the results only by observing the trajectory of body sway. Therefore, we attempted to obtain an error ellipse (probability ellipse) that covers the results of body sway, and to evaluate it quantitatively using three parameters: long diameter, declination, and eccentricity. The error ellipse (probability ellipse) is the result of calculating the elliptic equation from the variance–covariance matrix of a two-dimensional normal distribution from the body sway trajectory. In this article, the cumulative distribution function was set to 0.900, so that the ellipse was fitted to cover 90% of the trajectory range. By obtaining the basis vectors from the mean vectors and covariance vectors of the left–right and front–back oscillations of the body sway measurement results, we can obtain the long and short diameters and the declination along the axis. Declination was evaluated within the range of (−90°, 90°) to 0° midline. If the ellipse is tilted to the left, it is marked within the range (0°–90°), with a positive value, and if tilted to the right, it is marked within the range (−90°–0°), with a negative value.

**Figure 2:**
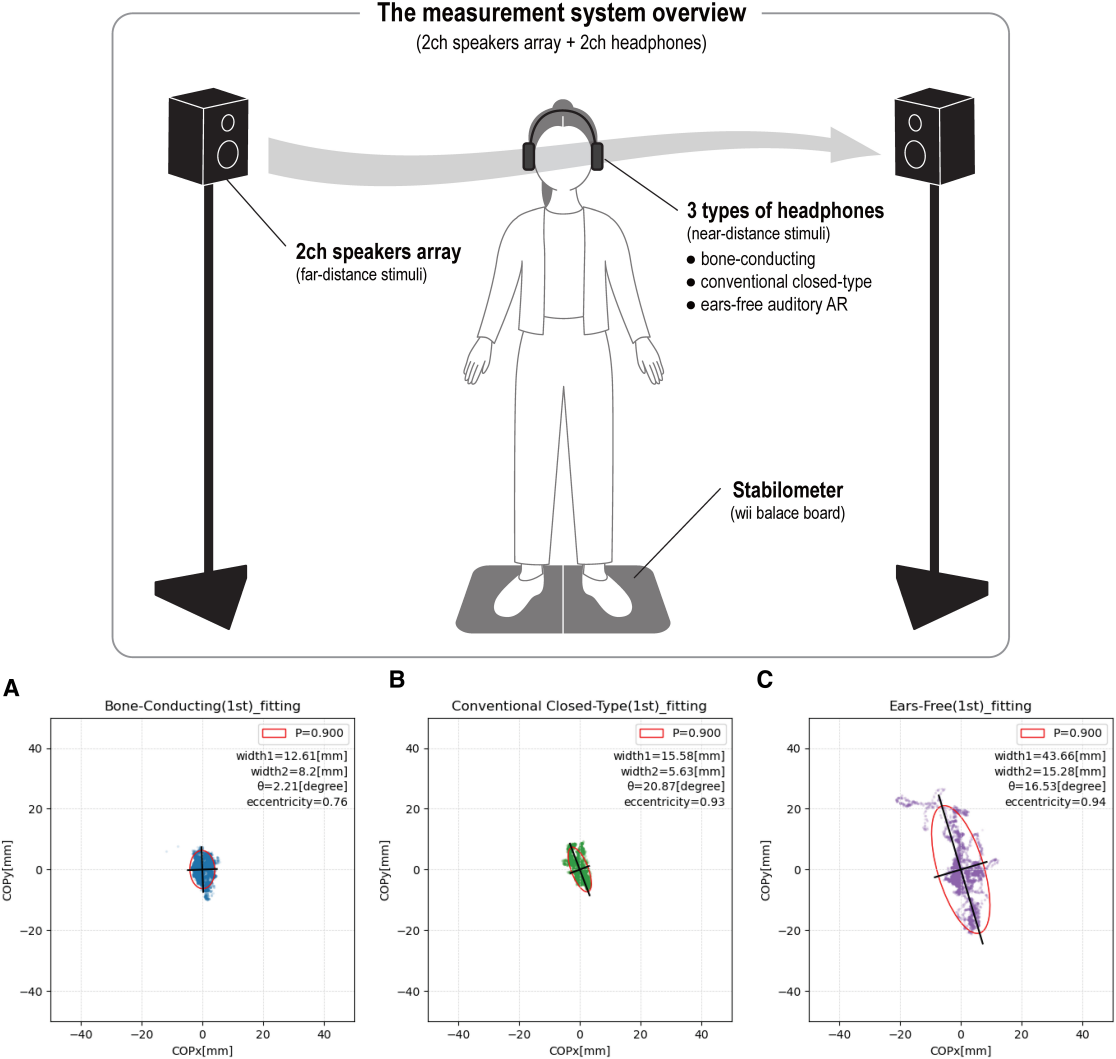
(**A**) Bone-conducting. (**B**) Conventional closed-type. (**C**) Ears-free.

Figure 2 is an example of fitting an error ellipse to the elementary data of body sway COP measurement results. The following graph shows the results of the body sway with the setting to 0 mm of the center.

In the example in Figure 2, when the ears-free headphones are worn, the ratio of the long to short diameters is the largest, resulting in an elongated ellipse. To evaluate the shape of this ellipse, the eccentricity *ε* was determined. The eccentricity ε of the ellipse can be expressed as follows, using the long diameter *a* and the short diameter *b*.

$$\varepsilon =\sqrt{\frac{a^{2}-b^{2}}{a^{2}}}.$$

Therefore, the closer the ellipse shape is to a circle, the smaller the value of the eccentricity. In Figure 2, the ears-free headphone has the largest eccentricity. The values are 0.76 for bone-conduction headphone (Figure 2A), 0.93 for closed-type headphone (Figure 2B), and 0.94 for ears-free headphone (Figure 2C), and the eccentricity becomes smaller in the order of ears-free, closed-type, and bone-conduction headphone. In other words, when wearing the ears-free headphone, the fitted ellipse has a large ratio of long to short diameter and an elongated sway range, which can be shown both in the figure and numerically.

The body sway can be evaluated based on the following parameters:

(1) Eccentricity *ε* of the ellipse.
(2) Long diameter *δ* of the ellipse.
(3) Declination *ϑ* of the long diameter.

We call this “the probability elliptic evaluation method” and proceed with the following analysis.

### 3.2 Evaluation

In the tables presented, “Background” is the condition without sound stimuli, and “Standard Stimuli” is the condition when stimuli were presented only with the array speakers. “1st trial” is the first of 10 panning sessions, and “2nd trial” is the second of 10 panning sessions. The “Effect of Stimuli” is the ratio of the “Background” and “Standard Stimuli”, or “1st trial” and “2nd trial” in the case of eccentricity and diameter, and the difference in the case of declination.

Table 2(a–c) present the results for means of Eccentricity *ε* (a), Diameter δ (b), and Declination ϑ (c) when the probability ellipse was fitted.

**Table 2:** Means and effects of stimuli about eccentricity, long diameter and declination.

(a) Mean eccentricity ɛ (#)		
① Background noise only	② Standard stimuli with Array speakers only	Array speakers: Effect of stimuli
$\varepsilon_{0}=0.87$	$\varepsilon_{s}=0.88$	$r_{l}=\frac{\varepsilon_{s}}{\varepsilon_{0}}=1.01\approx 1$

Standard stimuli with Array speakers +	1st trial	2nd trial	Effect of stimuli
③ Bone-conduction headphone	$\varepsilon_{1}=0.89$	$\varepsilon_{2}=0.85$	$\;r_{l}=\frac{\varepsilon_{2}}{\varepsilon_{1}}=0.96\approx 1$
④ Conventional closed-type headphone	$\varepsilon_{1}=0.89$	$\varepsilon_{2}=0.91$	$\;r_{l}=\frac{\varepsilon_{2}}{\varepsilon_{1}}=1.02\approx 1$
⑤ Ears-free headphone	$\varepsilon_{1}=0.89$	$\varepsilon_{2}=0.90$	$\;r_{l}=\frac{\varepsilon_{2}}{\varepsilon_{1}}=1.01\approx 1$

(b) Mean long diameter δ (mm)		
① Background noise only	② Standard stimuli with Array speakers only	Array speakers: Effect of stimuli
$\delta_{0}=22.28$	$\delta_{s}=22.17$	$r_{l}=\frac{\delta_{s}}{\delta_{0}}=1.00$

Standard stimuli with Array speakers +	1st trial	2nd trial	Effect of stimuli
③ Bone-conduction headphone	$\delta_{1}=23.62$	$\delta_{2}=20.09$	$\;r_{l}=\frac{\delta_{2}}{\delta_{1}}=0.85\approx 1$
④ Conventional closed-type headphone	$\delta_{1}=20.48$	$\delta_{2}=25.02$	$\;r_{l}=\frac{\delta_{2}}{\delta_{1}}=1.22\approx 1$
⑤ Ears-free headphone	$\delta_{1}=20.45$	$\delta_{2}=22.26$	$\;r_{l}=\frac{\delta_{2}}{\delta_{1}}=1.09\approx 1$

(c) Mean declination *θ* (°)		
① Background noise only	② Standard stimuli with Array speakers only	Array speakers: Effect of stimuli
$\vartheta_{0}=0.20^{\circ }$	$\vartheta_{s}=-0.92^{\circ }$	$\Delta \vartheta_{0\to s}=-1.12^{\circ }$

Standard stimuli with Array speakers +	1st trial	2nd trial	Effect of stimuli
③ Bone-conduction headphone	$\vartheta_{1}=1.22^{\circ }$	$\vartheta_{2}=-0.66^{\circ }\approx 0$	$\;\Delta \vartheta_{1\to 2}=-1.88^{\circ }$
④ Conventional closed-type headphone	$\vartheta_{1}=1.75^{\circ }$	$\vartheta_{2}=-0.03^{\circ }≅0$	$\;\Delta \vartheta_{1\to 2}=-1.78^{\circ }$
⑤ Ears-free headphone	$\vartheta_{1}=6.32^{\circ }$	$\vartheta_{2}=-0.17^{\circ }≅0$	$\;\Delta \vartheta_{1\to 2}=-6.49^{\circ }$
Declination angle ϑ of Ears-free headphone and Array speakers only condition.			
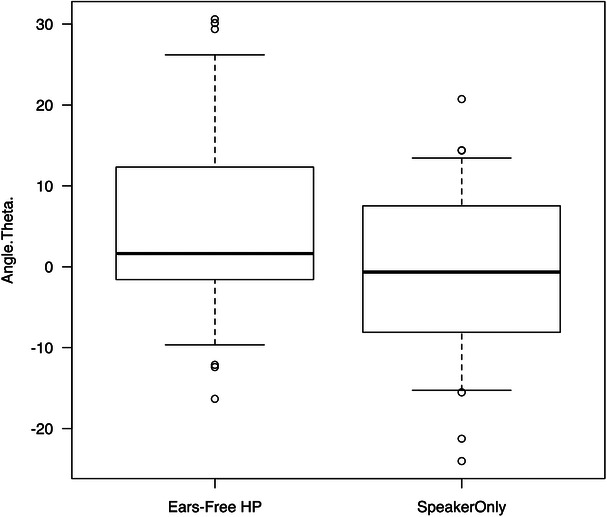			

(d) Mean lateral component ψ (mm)		
① Background noise only	② Standard stimuli with Array speakers only	Array speakers: Effect of stimuli
$\psi_{0}=-0.04≅0$	$\psi_{s}=0.03≅0$	$r_{l}=\frac{\psi_{s}}{\psi_{0}}=-0.09$

Standard stimuli with Array speakers +	1st trial	2nd trial	Effect of stimuli
③ Bone-conduction headphone	$\psi_{1}=0.30\approx 0$	$\psi_{2}=-0.35\approx 0$	$\;r_{l}=\frac{\psi_{2}}{\psi_{1}}=-1.17$
④ Conventional closed-type headphone	$\psi_{1}=0.31\approx 0$	$\psi_{2}=0.15\approx 0$	$\;r_{l}=\frac{\psi_{2}}{\psi_{1}}=0.47$
⑤ Ears-free headphone	$\psi_{1}=0.92\approx 1$	$\psi_{2}=-0.13\approx 0$	$\;r_{l}=\frac{\psi_{2}}{\psi_{1}}=-0.14$
Normal distribution graphs for the lateral components.			
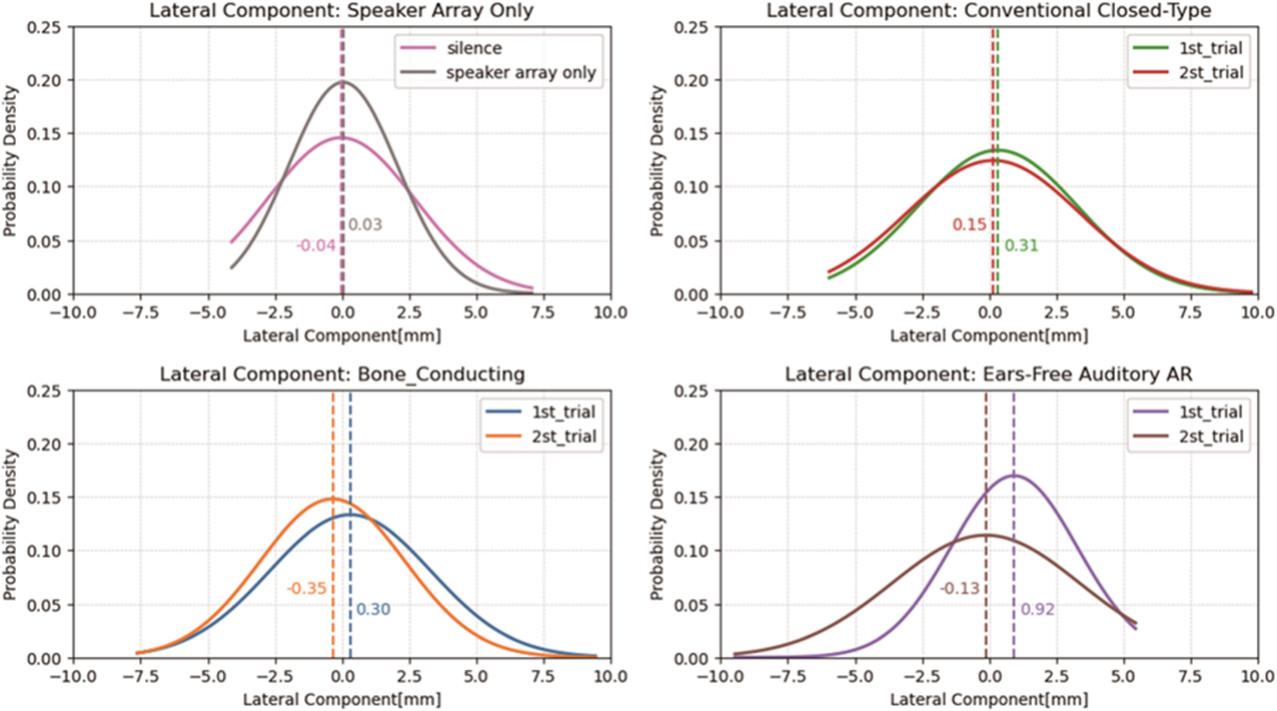			

Table 2(d) also shows the Lateral Component *Ψ* obtained by multiplying the sine of the declination by the radius of motion (half-length diameter). The “Effect of Stimuli” represents the difference between the first and second trials, whose normal distribution is shown below Table 2(d).

First, the “Eccentricity” *ε* was shown on Table 2(a): 0.89 and 0.85 for the first and second trials when wearing bone-conduction headphone, 0.89 and 0.91 with a conventional headphone, and 0.89 and 0.90 with the auditory AR headphone. The “Effect of Stimuli” was not significantly observed, and the ratios between the first and second trials are roughly equal to 1.00.

For “Long Diameter,” the bone-conduction headphones were 23.62 and 20.09 mm for the first and second trials, respectively; for the regular headphones, 20.48 and 25.02 mm; and for the auditory AR headset, 20.45 mm and 22.26 mm. As with the eccentricity, the ratio of the first to the second trial was almost equal to 1.00 and no significant change was observed.

The declination *θ* on Table 2(c): There is no significant difference between ② the “Array speakers only” condition and ③ the “Array speaker + Bone-conduction headphone” condition, and there is also no significant difference between ② the “Array speakers only” condition and ④ the “Array speaker + Closed-type headphone” condition.

There is a significant difference between ② the “Array speakers only” and ⑤ the “Array speaker + Ears-free headphone” condition in the first trial [“Array speakers only”: mean = −0.92°, SD = 11.09; “Ears-free headphone”: mean = 6.32, SD = 13.17, mean difference = −7.241363, paired *t*-test = −2.4917, df = 28, *p*-value = 0.019 (two-sided), 95% confidence interval (−13.19 to −1.29)]. The distribution of the difference between the two conditions does not have a significant deviation from the standard distribution [exact one-sample Kolmogorov−Smirnov test: *D* = 0.09, *p*-value = 0.94 (two-sided), Shapiro−Wilk normality test: *W* = 0.96, *p*-value = 0.33].

To evaluate the declination more rigorously, we obtained the lateral sway components to multiply by the sine of the declination and the radius of motion; the values in Table 2(d) show the mean of the lateral sway components and the graphs show the normal distribution on each situation.

We checked the values in Table 2(d) for ① the “Background noise only” condition. The value was −0.04 mm when no standard stimuli were presented. In the case of ② the “Standard stimuli with Array speakers only.” in which stimuli were presented only with the array speakers, the value was 0.03 mm. Both values are close to zero, and almost no swaying was observed. Conversely, in the case of ⑤ the “Array speaker + Ears-free headphone” condition, the lateral sway was the largest at 0.92 mm at the first trial, and it was observed to decrease significantly from the second trial.

The absolute value of “Effect of stimuli” is small for ⑤ the “Array speaker + Ears-free headphone” condition, which have the largest lateral component in the first trial, and increase in the order of conventional closed-type headphone and bone-conduction headphone.

The normal distribution graph also confirmed that the lateral body sway was largest for the ears-free headphone. The reason for this is explained in Section 4.1.

Without specific sound stimuli, naturally the l human body sways mostly in the longitudinal direction (4). Lateral sways are induced mostly by auditory stimuli and are estimated by the declination angle of the long diameter from the midline.

As described previously, a statistically significant difference in declination *θ* [Table 2(c)] was identified between ② the “Standard stimuli with Array speakers only” condition and ⑤ the “Array speaker + Ears-free headphone” condition. From this measurement, we have confirmed that the proximal auditory AR system works from the auditory periphery to the autonomic nervous system, and can be applied to vestibular rehabilitation.

## 4 Discussion

### 4.1 Virtual impact

We were able to confirm the effect of the panning stimulus presentation by evaluating the eccentricity *ε*, long diameter δ, declination ϑ, and the mean of each of the participants, especially the declination ϑ. In the case of eccentricity and long diameter, no significant effects were observed regardless of the stimuli presentation conditions. Conversely, in the case of the declination angle, significant differences were observed between the “Array speakers only” condition and the “Array speakers + Ears-free headphone” condition. The reason why this effect increases in the case of the “Array speakers + Ears-free headphone” condition is thought to be related to the transmission velocity and virtual impact that the participants would conceive during those trials. Array speakers are located physically apart from each other and auditory stimuli signal run rapidly on the pathway. Using the ears-free AR headphone, this virtual impact directly stimulates our inner-ear sensations and thus the largest reactions would be observed.

In this experimental system, there are two main types of sound transmissions: first, air transmission, in which air vibrations enter the ear canal through the auricle and vibrate the eardrum, and second, bone-conduction transmission, in which vibrations from the skull and soft tissue of the head are transmitted directly to the middle and inner ear. Based on this, we evaluated the speed of virtual sound image transfer using bone-conduction headphone, closed-type headphone, and ears-free headphone in a near-sighted auditory AR system as follows.

First, in the case of ears-free headphone, the sound image moves between distant array speakers without closing the auricle. The array speakers are placed 1 m to the left and 1 m to the right from the midline, so the distance *D* between the arrayed speakers is 2 m. The sound stimulus panning from left to right or right to left is scanned for 1 s, so the virtual speed of sound image movement for the ears-free headphone is ∼2 m/s.

Second, in the case of the closed-type, the sound source is located at the auricle, and the virtual sound image travel distance is shorter than that of the ears-free headphone. The National Institute of Advanced Industrial Science and Technology (AIST) of Japan reported that the head width of Japanese people is 15.33 cm. We add the length of the both auricles and assumed that the distance from the left auricle to the right auricle is 20 cm. We define this distance as *L*. The sound stimulus is scanned for 1 s, but the interval (*D* - *L*), which is the distance *D* between the array speakers minus the distance *L* between the auricles, is shorter than 1 s because no sound is heard in the closed-type auricle. If we assume that the stimulus is scanning for 0.5 s, the virtual speed of sound image movement for a closed-type headphone would be ∼0.4 m/s, which is slower than the speed for an ears-free headphone.

Finally, consider the bone-conduction headphone. It is easy to assume that the sound via bone-conduction headphones travels the same distance as via the ears-free headphone because the auricle is open. However, as mentioned previously, bone-conduction headphones vibrate the head directly and transmit stimuli to the middle and inner ears, so signals from bone-conduction headphones are likely to take precedence over those from array speakers. In addition, the speed of transmission from the skull to the middle and inner ears can be considered much faster than the speed of movement in air, since vibration is transmitted through a solid or liquid medium. Even if water were the only medium, the speed of sound would be 1,500 m/s, traveling a distance of about 3 cm to the outer ear in a time of 0.02 ms. If we consider this in terms of frequency, it is 50 kHz, which means that the sound moves at a frequency beyond the human audible range (20 Hz–20 kHz).

In other words, the distance that sound travels from the array speakers to the auricle (*D* − *L*) can be considered negligibly small when bone-conduction headphones are used. From this point, the travel distance of the virtual sound image is only the distance between the inner ears, which is approximately 12 cm, equal to the distance *L* of 20 cm between both auricles minus the length of both external auditory canals of 6 cm. Since the sound stimulus cans this distance for 0.5 s, the virtual sound image travel speed of the bone conduction headphones is about 0.24 m/s.

From here, the virtual momentum (impact) of each headphone is evaluated. Since sound has no weight, a “virtual impact model” was established and evaluated by applying a unit mass of 1 kg to the virtual sound image velocity.

Virtual impact (kg m/s) = unit mass (kg) × virtual sound image travel speed (m/s).

Hypothetically, for this measurement involving the human body, we multiplied the unit mass by 102 and used a mass of 100 kg for the evaluation. Multiplying the virtual sound image velocity of each headphone by 100 kg to obtain the virtual impact would be 24 kg/m/s for the bone-conduction type, 40 kg/m/s for the closed-type, and 200 kg/m/s for the ears-free headphone. Assuming no loss of kinetic energy as the virtual impact rushes into our ears, the highest impact on body sway would be for the ears-free headphone with a momentum of 200 kg m/s. The next highest impact would be for the closed-type headphone with 40 kg m/s, and the lowest impact for the bone-conduction headphone with 24 kg m/s of momentum. The ears-free headphone would have caused the body to sway because of the high virtual impact, and as a result, the declination angle of the ellipse fitted to the body sway trajectory would have been tilted significantly.

### 4.2 Prospects of further study

In the present experimental system, the researchers used healthy participants aged 19–22 years. The results showed that panning stimulation using a near-simultaneous auditory AR system induces body sway, and that continuous use of the system may attenuate reflexive and extrapyramidal sway of the body. This phenomenon is expected to be applied to the examination and rehabilitation of vestibular diseases and to the training of elderly people to hold a standing position by subliminal control of body swaying.

Conversely, since the rehabilitation program is still in the development stage, the measurements were conducted only on normal-hearing participants because it is necessary to ensure sufficient safety against falls and the like. Different results may be obtained for patients with dizziness or difficulty in standing.

In the future, we plan to ask patients with Parkinson's disease to cooperate with us to measure and evaluate their responses, and to study practical vestibular training methods.

Our system trains the vestibular organs by stimulating the autonomic nervous system through sound stimulation that dares the participant to break the upright position, thereby stimulating the body itself to maintain the upright position reflexively (subliminally) from a subconscious level. In this respect, it is completely different in principle from existing vestibular training (5–11). When a fine stimulus that dares the patient to lose balance is sent, the cerebellum reflexively tries to recover from it at the level of unconsciousness. Repeated stimulation of this kind is expected to make the body learn to recover easily even if the patient loses his or her balance.

Such physical information is also expected to be effectively utilized in machine learning. Generative AI, which is currently in vogue, exclusively analyzes textual data. However, in the midst of this boom, we would like to continue our efforts to develop new methods for diagnosing vestibular functions and training for healing by detecting extrapyramidal body movement data and directly analyzing the screams emitted by the body through rather old-fashioned machine learning that recognizes patterns in vector data of non-generative systems. We would like to continue to work on the development of new vestibular function diagnosis and training methods for healing.

## Data Availability

The original contributions presented in the study are included in the article/Supplementary Material, further inquiries can be directed to the corresponding author.
